# Biallelic *KCTD3* nonsense variant derived from paternal uniparental isodisomy of chromosome 1 in a patient with developmental epileptic encephalopathy and distinctive features

**DOI:** 10.1038/s41439-023-00250-z

**Published:** 2023-08-07

**Authors:** Keiko Shimojima Yamamoto, Ayumi Yoshimura, Toshiyuki Yamamoto

**Affiliations:** 1 Department of Transfusion Medicine and Cell Processing, Tokyo Women’s Medical University, Tokyo, 162-8666 Japan; 2 Institute of Medical Genetics, Tokyo Women’s Medical University, Tokyo, 162-8666 Japan; 3 Department of Pediatrics, Seirei Mikatahara General Hospital, Hamamatsu, 433-8558 Japan

**Keywords:** Uniparental disomy, Genetics research

## Abstract

A biallelic nonsense variant of the potassium channel tetramerization domain-containing protein 3 gene (*KCTD3*) [c.1192C>T; p.R398*] was identified in a patient with developmental epileptic encephalopathy with distinctive features and brain structural abnormalities. The patient showed isodisomy of chromosome 1, where *KCTD3* is located, and the father was heterozygous for the same variant. Based on these findings, paternal uniparental disomy was considered to cause the biallelic involvement of KCTD3.

Many disease-causing genes associated with developmental and epileptic encephalopathy (DEE) have been identified using whole-exome sequencing^1^. Most genes responsible for DEE are related to autosomal dominant traits, and their disease-causing variants are often identified as de novo occurrences^2^. In comparison, DEE-related genes with autosomal recessive traits have been observed to have a lower incidence^3^. This is because the autosomal recessive type of DEE often occurs due to homozygous disease-causing variants in consanguineous families.

The potassium channel tetramerization domain-containing protein 3 gene (*KCTD3*) was first identified as the gene responsible for a genetic disorder through clinical exome sequencing, and a homozygous truncating variant, NM_016121.5:c.1036_1073del [p.P346Tfs*4], was identified in a consanguineous family^4^. The same homozygous variant has been recurrently observed in multiple patients with hydrocephalus, seizures, global developmental delay, and abnormal brain structures^5^. Subsequently, an additional seven patients with biallelic *KCTD3* variants from mostly consanguineous families were reported by Faqeih et al.^6^. Among the seven reported patients, five patients from two consanguineous families shared the same p.P346Tfs*4 variant, but the other two patients from two different consanguineous families were homozygous for a novel nonsense variant, c.166C>T [p.R56*]. More recently, a novel nonsense variant, c.1261C>T [p.R421*], was further identified in a homozygous pattern^7^. All previously reported pathogenic *KCTD3* variants were related to loss of function. Although detailed clinical features were only available in the report by Faqeih et al.^6^, DEE, global developmental delay, and neuroradiological abnormalities were commonly observed in all reported patients. Thus, *KCTD3* was recognized as a DEE-related gene with autosomal recessive traits.

KCTD3 is a member of the KCTD family^8^. Several human KCTD genes are associated with neurodevelopmental disorders. The KCTD gene family commonly has a BTB domain but no transmembrane domain. *KCTD3* contains five WD repeats in addition to a BTB domain^9^. It has been reported that mouse Kctds and Hcn3 colocalize in brain regions and that Kctd3 increases the current density of Hcn3 by promoting the trafficking of Hcn3 protein to the cell membrane, as Hcn3 is a transmembrane protein^9^.

We recently encountered a patient with a biallelic *KCTD3* nonsense variant. The parents of this patient were not consanguineous, but the patient’s biallelic involvement was derived from uniparental disomy (UPD). This is the first case of a biallelic *KCTD3* variant derived from a patient with UPD.

The patient was a boy born at 40 weeks of gestation via normal vaginal delivery. His 32-year-old father was Peruvian, and his 28-year-old mother was Japanese. They had previously experienced a spontaneous abortion. At birth, his weight was 2994 g (25th~50th centile), his length was 50.0 cm (50th~75th centile), and his occipitofrontal circumference (OFC) was 33.5 cm (mean). He showed no asphyxia. As dilatation of the lateral ventricles was noted during pregnancy, brain magnetic resonance imaging (MRI) was performed 4 days after birth, and mild dilatation was still observed (not shown). The neonate was discharged on the same day. A health checkup at 1 month suggested brachycephaly. From the age of 4 months, he started to show episodes of tonic convulsions that continued for several minutes. A health checkup at 4 months suggested developmental delay, no head control and no eye tracking. The patient was admitted to our hospital for further examination. Generalized hypotonia and sensorineural hearing loss were also observed. Distinctive features included brachycephaly, a prominent forehead, deeply set eyes, a high-arched palate, micrognathia, low-set ears, bilateral clasped thumbs, and a small penis. His convulsions were determined to be focal seizures, and electroencephalography revealed multifocal spikes. After 6 months, a series of spasms and hypsarrhythmia were noted, and West syndrome was diagnosed. At 9 months, his weight was 7.6 kg (25th~50th centile), his length was 71.4 cm (90th~97th centile), and his OFC was 44.8 cm (75th~90th centile). However, the patient could still not control his head. Visual and auditory responses were not observed. Brain MRI findings showed abnormal brain structures with hypoplastic vermis of the cerebellum and dilatation of the lateral ventricles (Fig. 1). There was no finding of the Dandy–Walker variant.

**Fig. 1:**
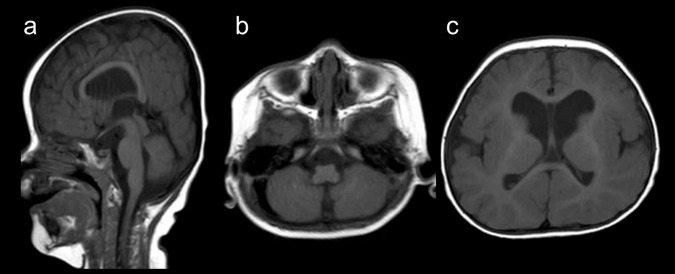
T1-weighted brain magnetic resonance imaging at 9 months. **a** A sagittal image shows brachycephaly and a thin corpus callosum. **b**, **c** Axial images show hypoplastic vermis of the cerebellum (**b**) and dilatation of the lateral ventricles associated with the cavum septum pellucidum.

To determine the genetic background of the patient, microarray-based comparative genomic hybridization was performed using SurePrint CGH + SNP 180 K (G4890A; Agilent Technologies, Santa Clara, CA, USA). The results showed no pathogenic copy number variation but did show a complete loss of heterozygosity (LOH) through chromosome 1 (Fig. 2a), indicating copy-neutral isodisomy. Under the suspicion of the presence of a homozygous pathogenic variant on chromosome 1, whole-exome sequencing was performed using SureSelect Human All Exon V6 (Agilent Technologies). The extracted FASTQ file was annotated using SureCall NGS Data Analysis Tool (Agilent Technologies). This study was performed in accordance with the Declaration of Helsinki, and requisite permission was obtained from the institutional ethics committee. Peripheral blood samples were collected from the patient and his parents after obtaining written informed consent from the family.

**Fig. 2:**
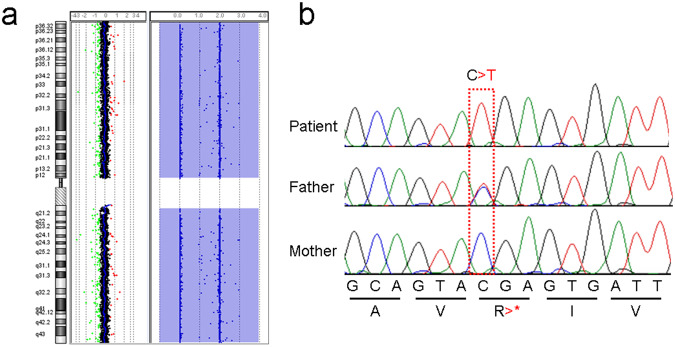
Results of the molecular analyses. **a** Results of chromosomal microarray testing for chromosome 1. The comparative genomic hybridization panel (left) and single-nucleotide polymorphism panel (right) indicate copy neutral but loss of heterozygosity in chromosome 1, respectively, suggesting complete isodisomy. **b** Electrophoresis images of Sanger sequencing. The patient shows a homozygous C>T variant, and his father is heterozygous for the variant. However, his mother had no variant.

After annotation, homozygous variants on chromosome 1 were analyzed. NM_016121.5 (KCTD3 exon13):c.1192C>T [p.R398*] was identified as a homozygous loss-of-function variant on chromosome 1. Variants in all chromosomal regions were analyzed; however, no other possible pathogenic variants were identified. Subsequent Sanger sequencing identified the same variant in his father in the heterozygous state (Fig. 2b). This variant was identified as rs764361600 in the SNP database (https://www.ncbi.nlm.nih.gov/snp/), and the gnomAD browser (http://www.gnomad-sg.org/) revealed a global allele frequency of 0.000003979 (1/251294). Currently, this variant is not included in ClinVar (https://www.ncbi.nlm.nih.gov/clinvar/). According to the American College of Medical Genetics and Genomics guidelines^10^, this variant can be scored as PVS1 and PM2, indicating it is “likely pathogenic”.

The clinical features of the present patient were compatible with those of previously reported patients with KCTD3-related DEE, including global developmental delay and brain structural abnormalities. Although detailed clinical features could only be compared with those reported by Faqeih et al., the generalized hypotonia and distinctive features in the present patient were frequently observed in their patients^6^. Therefore, a molecular diagnosis of *KCTD3*-related DEE was established. Owing to the biallelic involvement of the *KCTD3* nonsense variant, the null function of KCTD3 could be considered a mechanism.

Although it is rare, the biallelic involvement of autosomal recessive trait genes derived from UPD has been reported in some studies. The contactin 2 gene (*CNTN2*) is located on 1q32.1. Biallelic involvement of the *CNTN2* truncating variant (p.T958Tfs*17) was identified in a patient with neurodevelopmental impairment and focal seizures^11^. The patient had a segmental LOH on chromosome 1. Further analyses confirmed maternal heterodisomy. A truncating variant existed in the LOH region. Another example is the biallelic variant in the dedicator of cytokinesis 7 gene (*DOCK7*; p.A1798Lfs*59), located in the segmental UPD of chromosome 1, which has been identified in a patient with DEE^12^. Similar to the case above, the truncating variant was located in a maternally derived LOH. In these two cases, the segmental UPD was of maternal origin, indicating trisomy rescue because most of the aneuploidies were derived from chromosomal nondisjunction in oocytes.

In the present case, chromosome 1 exhibited complete isodisomy. Complete isodisomy is the predominant UPD type observed in the largest chromosomes, and paternal UPD is enriched in isodisomy^13^. Although the age of the mother of the present patient was not advanced, it is known that chromosomal nondisjunction increases with maternal age and that advanced maternal age is related to UPD. Therefore, the increased ratio of paternal isodisomy is generally the result of maternal nondisjunction, and monosomy rescue is the mechanism. Although the father of the present patient was a heterozygous carrier of the *KCTD3* variant, the frequency of UPD is quite low, and the risk of recurrence in this family is insignificant. Thus, the molecular diagnosis described in this study would be beneficial for this family in terms of genetic counseling.

## Data Availability

The relevant data from this Data Report are hosted at the Human Genome Variation Database at 10.6084/m9.figshare.hgv.3312.
